# Precision Medicine: Applied Concepts of Pharmacogenomics in Patients with Various Diseases and Polypharmacy

**DOI:** 10.3390/pharmaceutics13020197

**Published:** 2021-02-02

**Authors:** Veronique Michaud, Jacques Turgeon

**Affiliations:** 1 Precision Pharmacotherapy Research and Development Institute, Tabula Rasa Health Care, Lake Nona, Orlando, FL 32827, USA; vmichaud@trhc.com or; 2 Faculty of Pharmacy, Université de Montréal, Montréal, QC H3C 3J7, Canada

Over the last century, the process of choosing medications to treat certain diseases has evolved significantly. In the early days of our pharmacological armamentarium, prescribers were selecting medications mostly by Preference whenever more than one option was available. With the venue of evidence-based medicine and the surge of clinical trials, drug selection became more standardized among prescribers as they paid more attention to patient characteristics and disease treatment guidelines. This brought us to the era of Personalized treatments. This is not to say that physicians were not paying attention to each patient prior this time, but as of then, drug selection was established on more sound and solid clinical evidence, running away from the “one-size-fits-all” approach.

On 16 April 1999, a short article appeared in *The Wall Street Journal* entitled “Genetic Mapping Ushers In New Era Of Profitable Personal Medicines”. At this time, the public was introduced to the term “Personalized Medicine”, and the article, written by Robert Langreth and Michael Waldholz, described the formation of the Single Nucleotide Polymorphism Consortium, an initiative leading to a large collaboration between several pharmaceutical companies [1]. This occurred as the Human Genome Project, launched in 1990, was to reach completion 4 years later [2,3]. Although the term “Personalized” was used at that time, several scientists now prefer to describe the use of genetics and other technologies to established treatment as “Precision” pharmacotherapy. Eventually, the use of preemptive testing favors more Predictive, Preventive, and Participatory medicine or pharmacotherapy, such that several versions of P(N) Medicine or P(N) Pharmacotherapy toponomy have been proposed [4].

In this colligated Special Issue of *Pharmaceutics* on “Precision Medicine: Applied Concepts of Pharmacogenomics in Patients with Various Diseases and Polypharmacy”, our objective is to offer the reader a series of articles that describe the concept of “Precision Medicine”, discuss its implementation process and limitations, demonstrate its value by illustrating some clinical cases, and open the door to new and more sophisticated techniques and applications.

In their review, Malsagova et al. lead the reader through the general concept of pharmacogenomics (PGx) and related issues of PGx testing efficiency, personal data security, and health safety at a current clinical level. The authors present a short history of PGx, describe various drug-metabolizing enzyme phenotypes, and illustrate the PGx testing cycle. They identify most relevant conditions and drugs where PGx testing could be applicable and also provide a list of PGx companies and services [5].

In a second article, Tata et al. describe the difficulties encountered in the implementation of PGx testing in sub-Saharan African countries. The authors recognize that PGx testing can significantly improve healthcare delivery considering the high incidence of communicable diseases, the increasing incidence of non-communicable diseases, and the high degree of genetic diversity in these populations. Among the limitations identified, the authors discuss under-resourced clinical care logistics, a paucity of PGx clinical trials, scientific and technical barriers to genotyping, and socio-cultural as well as ethical issues regarding healthcare stakeholders [6].

Third, Li et al. discuss the challenges encountered while trying to link PGx information on drug safety and efficacy in ethnic minority populations. They bring to light the notion that several clinical studies on PGx markers and related drug dose adjustments are often not conducted in diverse ethnic populations. To address this challenge, they initiated a bioinformatic project where PGx information is gathered from drug labels, extracted data on the allele frequency information of genetic variants in ethnic minority groups, and collected published research articles on PGx biomarkers to construct a new PGx database [7].

In the “applied section” of this Special Issue, six articles are presented to illustrate the clinical application and relevance of PGx testing and “Precision Medicine” for the diagnosis and treatment of various diseases under various conditions. Almenar-Pérez et al. present the study, “Impact of polypharmacy on candidate biomarker miRNomes for the diagnosis of fibromyalgia and myalgic encephalomyelitis/chronic fatigue syndrome: striking back on treatment”. Their study demonstrates that miRNomes could help refine PGx/pharmacoepigenomic analysis to elevate future personalized and precision medicine programs in the clinic [8]. Wigle et al. provide a focused review of 5-fluorouracil (5-FU) metabolism and efforts to improve predictive dosing through screening for dihydropyrimidine dehydrogenase (DPD) deficiency, a single enzyme largely responsible for the metabolism of 5-FU. Using a patient case related to an orthotopic liver transplant recipient, they highlight some limitations of PGx testing but suggest that such case supports the development of robust multimodality precision medicine services [9]. Michaud et al. provide compelling results on the role of glutathione S-transferase A1 (*GSTA1*) gene variants on busulfan oral clearance in a population of patients undergoing hematopoietic stem cell transplantations. They demonstrate that homozygote patients for GSTA1*B/*B exhibit much lower busulfan oral clearance than patients with a *A/*A genotype: after the first standard dose, 2/3 of GSTA1*B/*B patients had plasma levels above the therapeutic levels [10]. In their review, Suntsov et al. discuss how an individual’s genotype could affect their response to therapy, as well as how genetic polymorphisms in CYP450 and other enzymes are crucial for affecting the metabolic profile of drugs used for the treatment of chronic lower back pain. They suggest that implementation of gene-focused pharmacotherapy has the potential to deliver select, more efficacious drugs and avoid unnecessary polypharmacy-related adverse events in many painful conditions [11]. Buendia et al. report on the clinical value of PGx in special populations such as children undergoing liver transplantation. Their study demonstrates that the frequency of CYP3A5*1 expression for recipients was 37.1% and was 32.2% for donors. Patients who received an expresser organ showed a lower concentration/dose ratio, especially in the 90 days following the surgery. They conclude that the role of each polymorphism is different according to the number of days after the transplant. They also suggest that such polymorphism be considered to optimize the benefits of tacrolimus therapy during the post-transplant induction and maintenance phases [12]. Finally, in their study, Salvador-Martin et al. aimed to identify PGx markers that could predict early response to anti-tumor necrosis factor (TNF) drugs in pediatric patients with inflammatory bowel disease. They characterized whole-gene expression profiles from the total RNA of their patients and demonstrated overexpression of FCGR1A, FCGR1B, and GBP1 in non-responders to treatment [13].

We are convinced that this Special Issue on “Precision Medicine” will provide clinicians and scientists a perspective on the potential of PGx. We have paid special attention to colligate articles addressing implementation, limitations, applicability, and value, using clinical cases to inspire the scientific community in future development around precision medicine.
